# The correlation of anxiety and depression with C3 and C4 Levels and systemic lupus erythematosus activity

**DOI:** 10.1186/s12888-023-05285-8

**Published:** 2023-10-26

**Authors:** Hamzah Shatri, Purnamandala, Rudy Hidayat, Robert Sinto, Alvina Widhani, Rudi Putranto, Rr Dyah Purnamasari, Eka Ginanjar, Chyntia Olivia Maurine Jasirwan

**Affiliations:** 1 Division of Psychosomatic and Palliative, Department of Internal Medicine, Faculty of Medicine, Universitas Indonesia/ Cipto Mangunkusumo National Referral Hospital, Jakarta, Indonesia; 2 Department of Internal Medicine, Faculty of Medicine, Universitas Indonesia/ Cipto Mangunkusumo National Referral Hospital, Jakarta, Indonesia; 3 Division of Rheumatology, Department of Internal, Faculty of Medicine, Universitas Indonesia/ Cipto Mangunkusumo National Referral Hospital, Jakarta, Indonesia; 4 Division of Tropical and Infectious Diseases, Department of Internal Medicine, Faculty of Medicine Universitas Indonesia/ Cipto Mangunkusumo Referral National Hospital, Jakarta, Indonesia; 5 Division of Allergy and Clinical Immunology, Department of Internal Medicine, Faculty of Medicine, Universitas Indonesia/ Cipto Mangunkusumo National Referral Hospital, Jakarta, Indonesia; 6 Division of Endocrinology Metabolism and Diabetes, Department of Internal Medicine, Faculty of Medicine, Universitas Indonesia/ Cipto Mangunkusumo National Referral Hospital, Jakarta, Indonesia; 7 Division of Cardiology, Department of Internal Medicine, Faculty of Medicine, Universitas Indonesia/ Cipto Mangunkusumo National Referral Hospital, Jakarta, Indonesia; 8 Division oh Hepatobiliary, Department of Internal Medicine, Faculty of Medicine, Universitas Indonesia/ Cipto Mangunkusumo National Referral Hospital, Jakarta, Indonesia

**Keywords:** Anxiety, Depression, SLE, HADS, C3, C4, Complement, Mex-SLEDAI

## Abstract

**Background:**

Anxiety and depression are psychosomatic disorders that are frequently observed in chronic conditions such as systemic lupus erythematosus (SLE). Anxiety and depression can be induced by immunological and neurotransmitter dysregulation, which is characterized by hypothalamic–pituitary–adrenal (HPA) axis dysfunction, production of proinflammatory cytokines, and activation of complement in the blood, such as C3 and C4. The causes of anxiety and depression in SLE are complex, ranging from neuropsychiatric involvement to drug adverse effects. Detecting anxiety and depression symptoms in SLE patients is critical to preventing disability from impacting quality of life.

**Objective:**

To assess the relationship between anxiety and depression symptomatology, SLE disease activity with levels of C3 and C4 in Cipto Mangunkusumo National Hospital.

**Methods:**

This study used a cross-sectional design. The study included 120 SLE patients from Cipto Mangunkusumo National Hospital, aged 18 to 60 years. All patients were requested to complete a Hospital Anxiety and Depression Scale (HADS) questionnaire to assess their anxiety and depression symptoms. Subjects with anxiety and depression were assessed for disease activity using the Mexican Systemic Lupus Erythematosus Systemic Disease Activity (Mex-SLEDAI), and blood samples were collected to test complement C3 and C4 levels. Spearman's correlation test was used to examine the relationship between HADS scores, Mex-SLEDAI, and C3 and C4 levels.

**Results:**

The results of the study showed a very weak statistically significant negative correlation between anxiety symptoms based on HADS and C3 levels (*r* = -0.189; *p* = 0.038) and a weak correlation between anxiety symptoms and C4 levels (*r* = -204; *p* = 0.026). Depressive symptoms based on HADS revealed a very poor connection and no statistical significance with levels of C3 (*r* = -0.056; *p* = 0.546) and C4 (*r* = -0.068; *p* = 0.461). Anxiety (*r* = 0.06; *p* = 0.173) and depression (*r* = 0.031; *p* = 0.753) symptoms have a weak and insignificant positive connection with SLE activity.

**Conclusion:**

C3 and C4 serum levels appeared to decrease when the presence of anxious symptoms increased. There was no significant correlation in SLE disease activity between anxious and depressed patients.

## Introduction

The two most common manifestations of psychological problems are anxiety and depression. Anxiety disorders are the most frequent psychiatric disorder in the United States, affecting 40 million adults over the age of 18, or 18.1% of the population each year [1]. According to the World Health Organization (WHO), depression is the most prevalent cause of disability in 2015, affecting quality of life [2]. Depression and anxiety are psychological stressors that have been linked to several chronic inflammatory disorders, including systemic lupus erythematosus (SLE). Anxiety and depression are also indications of neuropsychiatry in SLE (NPSLE), which occurs when the inflammation in SLE worsens [3, 4].

SLE is an autoimmune illness with variable symptoms. There are various aggravating factors that impede remission and trigger relapse. Viral infections, UV light, medications, and hormones have all been studied as potential causes of SLE activity [5]. Based on the Hospital Anxiety and Depression Scale (HADS) evaluation, Zhang et al. discovered that anxiety problems were present in 40% of SLE patients. This is consistent with the findings of Henly et al.'s study, which show that anxiety is more influenced by external factors [6]. This contrasts with the occurrence of depression in SLE, where the HADS assessment revealed a 30% prevalence [7].

There is still no clear and defined pathogenesis of depression and anxiety in SLE. Various studies suggest inflammation, social factors, and medications influence mental issues in SLE patients. Kenna et al. discovered that steroid doses greater than 40 mg of prednisone can cause mood problems [8]. Immune dysregulation is also present in depression and anxiety, defined by hypothalamic–pituitary–adrenal (HPA) axis dysfunction and the release of pro-inflammatory cytokines. One common immunity dysregulation in SLE is complement activation. Complement activation is one indicator of inflammation since there was an increase in C3 and C4 levels in a person experiencing acute psychological stress [9].

In SLE patients, reduced complement serum level due to severe inflammation is one predictor of relapse. Gandino et al. found that in SLE patients with fluctuating complement levels, the frequency of lupus glomerulonephritis was higher than in individuals with normal complement, who had fewer hematological abnormalities and higher anti-dsDNA levels. We hypothesized that disease activity, which should be correlated to inflammation, including in Blood Brain Barrier or Central Nervous System, may contribute to occurrence of anxiety or depression in SLE patients. Immune system activation in SLE correlated to low C3 and C4 levels. So, the main goal of this study is to determine a correlation between anxiety and depression and complement serum levels in SLE patients. This study also looked at the relationships between anxiety and depression and SLE activity [10, 11].

## Methods

### Study design

This study used a cross-sectional design. All patients with SLE from the Internal Medicine outpatient clinic at Cipto Mangunkusumo National Hospital were included in the study. Based on inclusion and exclusion criteria, the sample was drawn using the consecutive sampling method. SLE patients using less than 40 mg of prednisone equivalent per day were enrolled in this study. We exclude patients consuming prednisone 40 mg or more because there was finding Nishimura et. al [12]. that it can contribute to psychiatric conditions.

### Eligibility criteria

The inclusion sample ranged in age from 18 to 60 years old and could both read and write. Patients were excluded if they had an acute infection for SLEs that lasted more than 7 days, a chronic infection (pulmonary tuberculosis, CMV, systemic fungus, HIV), steroid consumption of more than 40 mg prednisolone equivalent per day, a history of NPSLE, a history of psychopharmacology administration, or a history of very severe MEX SLEDAI.

### Variables

The HADS is a scale used to assess the degree of anxiety and depression with measurement using a 0—21 score questionnaire. C3 and C4 levels which are acute phase complements assessed in SLE patients are measured using enhanced polyethylene glycol (PEG). MEX SLEDAI instrument to measure the degree of SLE disease activity was measured based on anamnesis (psychiatric disorders, seizures, limb weakness, sensory disturbances), physical examination (motor strength, cranial nerves, vasculitis, muscle pain, joint pain, effusion, rash, oropharyngeal ulceration, alopecia, pericardial and pleural friction rub, rebound tenderness), and laboratory (peripheral complete blood, type count, reticulocytes, urinalysis, creatinine and creatinine kinase (CK)). We preferred MEX-SLEDAI to SLEDAI because of it is easier to used and has relatively good reliability and validity.

### Data collection

All subjects who got information signed an informed consent form. Furthermore, sociodemographic data was collected, which included filling out the HADS questionnaire, which was aided so that respondents had the same understanding of the questions, calculating the MEX-SLEDAI score, and supporting examinations in the form of blood tests performed on the same day without the need for fasting. Complete blood, reticulocytes, creatinine, CK, ureum, and complement levels (C3 and C4) were all measured through the subject's veins. In addition, a urinalysis examination was performed.

C3 and C4 levels were measured by using The BN ProSpec® System. It is using nephelometric technology to detect various markers, including C3 and C4 levels. The principle of measurement is measure of scattered light intensity in a fixed angle of 13—24 degrees. The samples collected were diluted with 1:1 comparison and put into test tube. We use standard reagent from the kit and measurement was done in 37 ± 1.5 °C [13].

### Statistical analysis

Data were analyzed using Statistical Analysis Software Package for Windows (SPSS, IBM, USA) version 25.0 software. If the data were normally distributed, a Pearson correlation test was applied to examine the connection between HADS scores, C3 and C4 levels, and MEX-SLEDAI scores in SLE patients with depression. The Spearman correlation test was applied if the data distribution was not normal.

## Results

The total number of research participants was 120, with the characteristics presented in Table 1. The patients' median age was 31 years, and they were all female. The median steroid dosage among the 120 study participants was 16 mg per week of methylprednisolone. Furthermore, the median MEX SLEDAI value for SLE activity was 6, indicating modest activity in 120 patients. Anxiety was identified in 35 percent of 120 individuals, with 22.5% having mild symptoms, 12.5% having moderate symptoms, and 0.1% having severe symptoms. While 27% of participants reported depressed symptoms, 20% reported mild symptoms, 7% reported moderate symptoms, and 0.1% reported severe symptoms. In addition, 87% of SLE patients who used DMARD used hydroxychloroquine, 66% used mycophenolate, 22% used azathioprine, and 9% used cyclosporine. 48 persons (40%) were found to be C3 deficient, while 15 people (12.5%) were found to be C4 deficient.

**Table 1:** Subject characteristics

Subject Characteristics	*n* = 120
Age (years), median (IQR)	31 (24–37)
Gender, n(%)	
Women	120 (100%)
Marital Status	
Married	63 (52.5%)
Unmarried	53 (44.2%)
Divorced	4 (3.3%)
Steroid dose (mg/week), median (IQR)	16 mg (0–28 mg)
Lupus activity (MEX SLEDAI), median (IQR)	6 (0–11)
HADS A score (Anxiety)	
Mild (8–10)	27 (22,5%)
Moderate (11–15)	15 (12,5%)
Severe (16–21)	1 (0,1%)
HADS D score (Depression)	
Mild (8–10)	24 (20%)
Moderate (11–15)	9 (7%)
Severe (16–21)	1 (0,1%)
Organ Involvement, n (%)	
Mucocutaneous	116 (96%)
Musculoskeletal	114 (95%)
Hematology	58 (48%)
Kidney	61(50%)
Serositis	6 (5%)
DMARD	
Azathioprine	22 (18%)
Cyclosporine	9 (7%)
HCQ	87 (72%)
Mycophenolate	66 (55%)
MTX	0 (0%)
Tacrolimus	1(0.8%)
Complement	
C3 < 90	48 (40%)
C4 < 12	15 (12.5%)

*DMARD* Disease-Modifying Antirheumatic Drugs, *HADS A* Hospital Anxiety and Depression Scale for anxiety, *HADS-D* Hospital Anxiety and Depression Scale for depression, *HCQ* Hydroxychloroquine, *MTX* Methotrexate, *C3* complement 3, *C4* complement 4

We also analyse the difference of baseline characteristics between decrease complement group and normal complement group, both C3 and C4. We find significantly higher hematology and kidney involvement in decreased C3 levels group (Table 2).

**Table 2:** Baseline characteristics across C3 complement levels group

Variables	Decrease C3 level, *n* = 48	Normal C3 Level, *n* = 72	*P* value
Age (years), median (IQR)	29 (15,5)	31 (13,5)	0,69
Marital Status			
Married	26 (54,1%)	37 (51,4%)	0,304
Unmarried	20 (41,67%)	33 (45,8%)	
Divorced	2 (4,17%)	2 (2,78%)	
Steroid duration (week), median (IQR)	16 (23)	16 (31)	0,736
Lupus activity (MEX SLEDAI), median (IQR)	8,5 (8,75)	6 (6)	0,057
HADS A score (Anxiety), median (IQR)	6 (5)	5 (5)	0,157
HADS D score (Depression), median (IQR)	6 (4)	6(2)	0,406
Organ Involvement, n (%)			
Mucocutaneous	46 (95,8%)	70 (97,2%)	1
Musculoskeletal	45 (93,8%)	69(95,8%)	0,68
Hematology	24 (50%)	34 (47,2%)	0,037
Kidney	30 (62,5%)	31 (43%)	0,037
Serositis	5 (10,4%)	4 (5,5%)	0,481
DMARD			
Azathioprine	12 (25%)	10 (13,9)	0,123
Cyclosporine	2 (4,2%)	7 (9,7%)	0,313
HCQ	34 (70,8%)	53 (73,6%)	0,738
Mycophenolate	28 (58,3%)	38 (52,7%)	0,549
MTX	0	0	NA
Tacrolimus	0	1 (1,4%)	1

*DMARD* Disease-Modifying Antirheumatic Drugs, *HADS A* Hospital Anxiety and Depression Scale for anxiety, *HADS-D* Hospital Anxiety and Depression Scale for depression, *HCQ* Hydroxychloroquine, *MTX* Methotrexate, *IQR* Inter quartile range

From Table 3 below we can see the difference of patient characteristics according C4 levels. Age is the only variable that has statistically significant differences (higher in decrease C4 group). All subjects are women, and there is no MTX user in our study.

**Table 3:** Baseline characteristics across C4 complement levels group

Variables	Decrease C4 level, *n* = 16	Normal C4 Level, *n* = 104	*P* value
Age (years), median (IQR)	37 (15,25)	30 (14)	**0,024**
Marital Status			
Married	11 (68,8)	52 (50)	0,722
Unmarried	4 (25)	49 (47,1)	
Divorced	1 (6,25)	3 (2,9)	
Steroid duration (week), median (IQR)	14 (27)	16 (28)	0,829
Lupus activity (MEX SLEDAI), median (IQR)	10 (8,75)	6 (7)	0,379
HADS A score (Anxiety), median (IQR)	6 (4,5)	5 (4,75)	0,558
HADS D score (Depression), median (IQR)	5 (3,75)	6 (3)	0,763
Organ Involvement, n (%)			
Mucocutaneous	15 (93,75)	101 (97,11)	0,485
Musculoskeletal	16 (100)	98 (94,23)	1
Hematology	7 (43,75)	51 (49)	0,694
Kidney	8 (50)	53 (50,9)	0,943
Serositis	1 (6,67)	8 (7,69)	1
DMARD			
Azathioprine	3(18,7)	19 (18,3)	1
Cyclosporine	2 (12,5)	7 (6,73)	0,343
HCQ	13 (81,3)	74 (71,2)	0,552
Mycophenolate	9 (56,3)	57 (54,8)	0,914
MTX	0	0	NA
Tacrolimus	0	1	1

*HADS-D* Hospital Anxiety and Depression Scale for depression, *HCQ* Hydroxychloroquine, *MTX* Methotrexate, *IQR* Inter quartile range

### Correlation of HADS-Anxiety with C3 levels and C4 levels

**Fig. 1:**
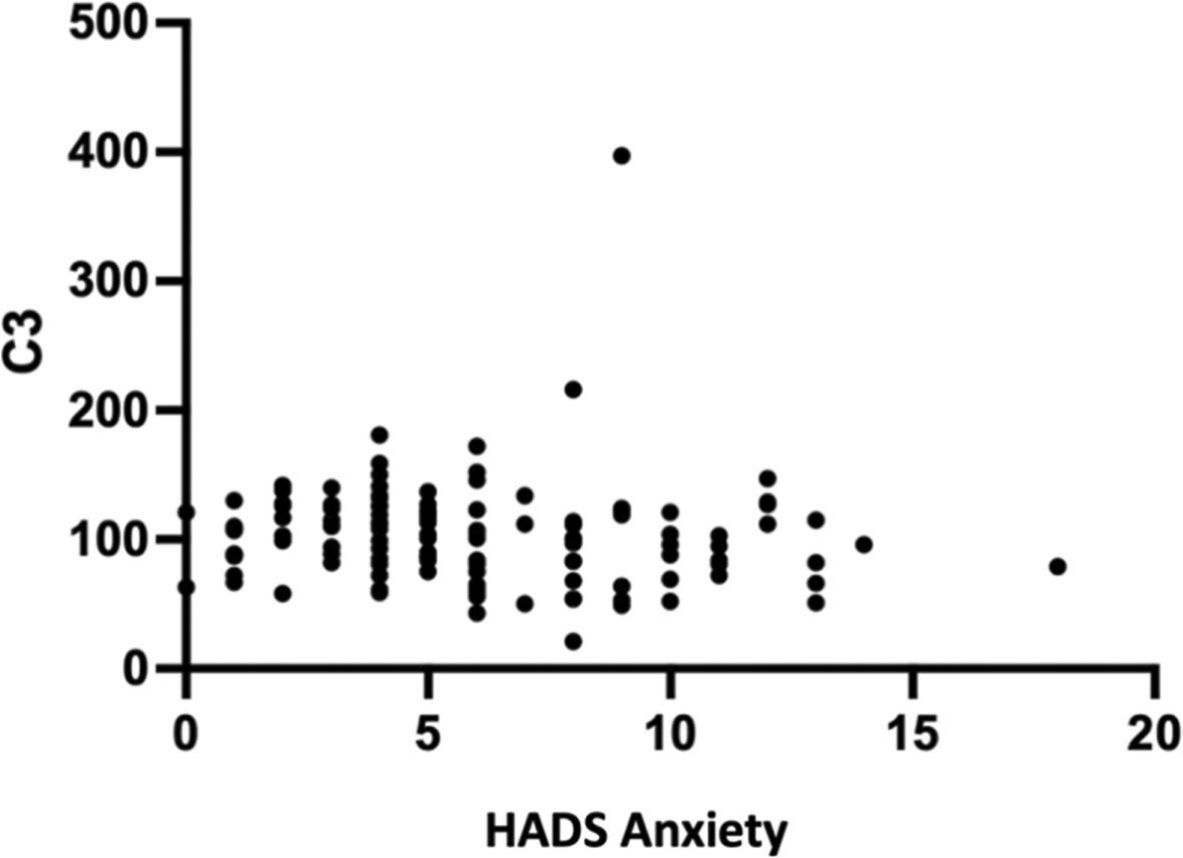
HADS correlation graph of anxiety with C3 levels

**Fig. 2:**
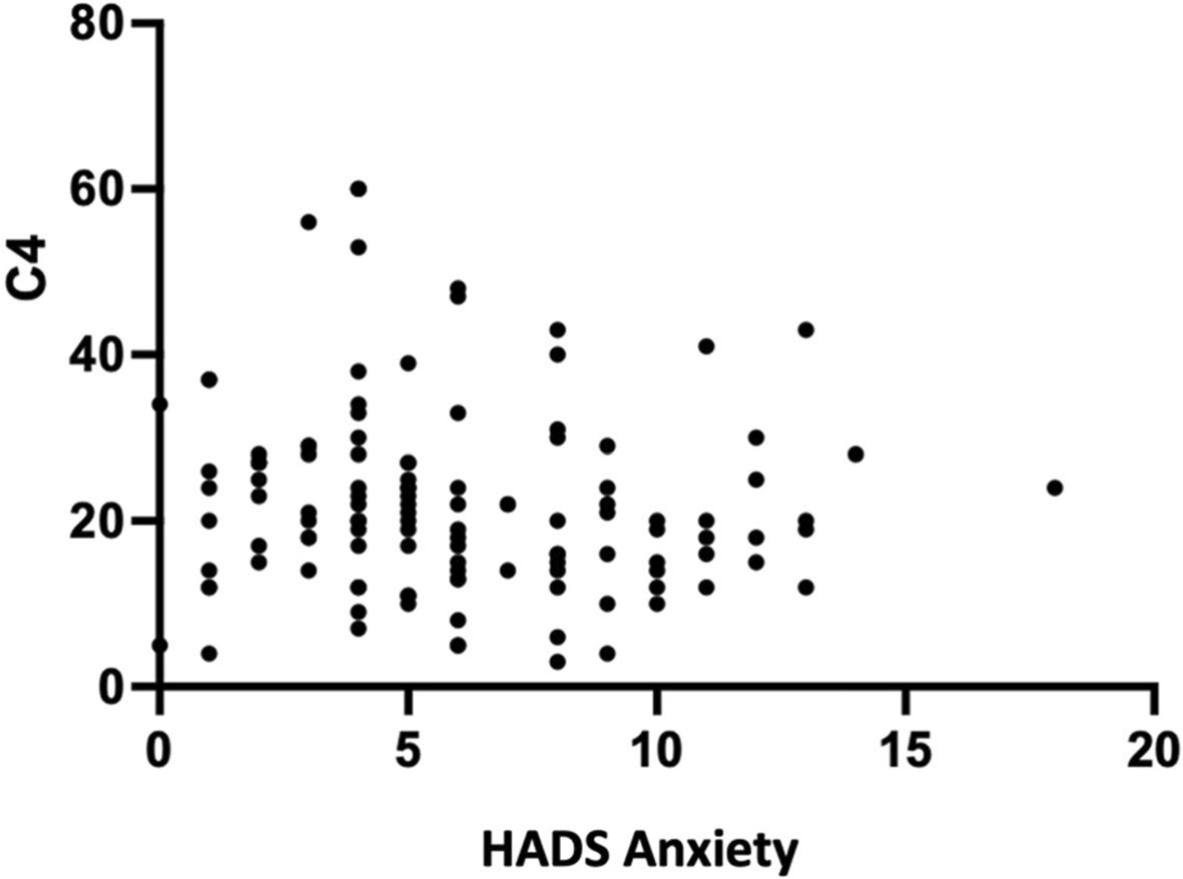
HADS correlation graph of anxiety with C4 levels

The Spearman correlation test revealed a statistically weak negative correlation between HADS anxiety and C3 levels (*r* = -0.189; *p* = 0.038). According to Fig. 1, the correlation coefficient number is negative. The correlation between HADS anxiety and C4 levels was small but significant (*r* = -0,185; *p* = 0.043), as illustrated in Fig. 2.

### Correlation of HADS-Depression with C3 and C4 levels

**Fig. 3:**
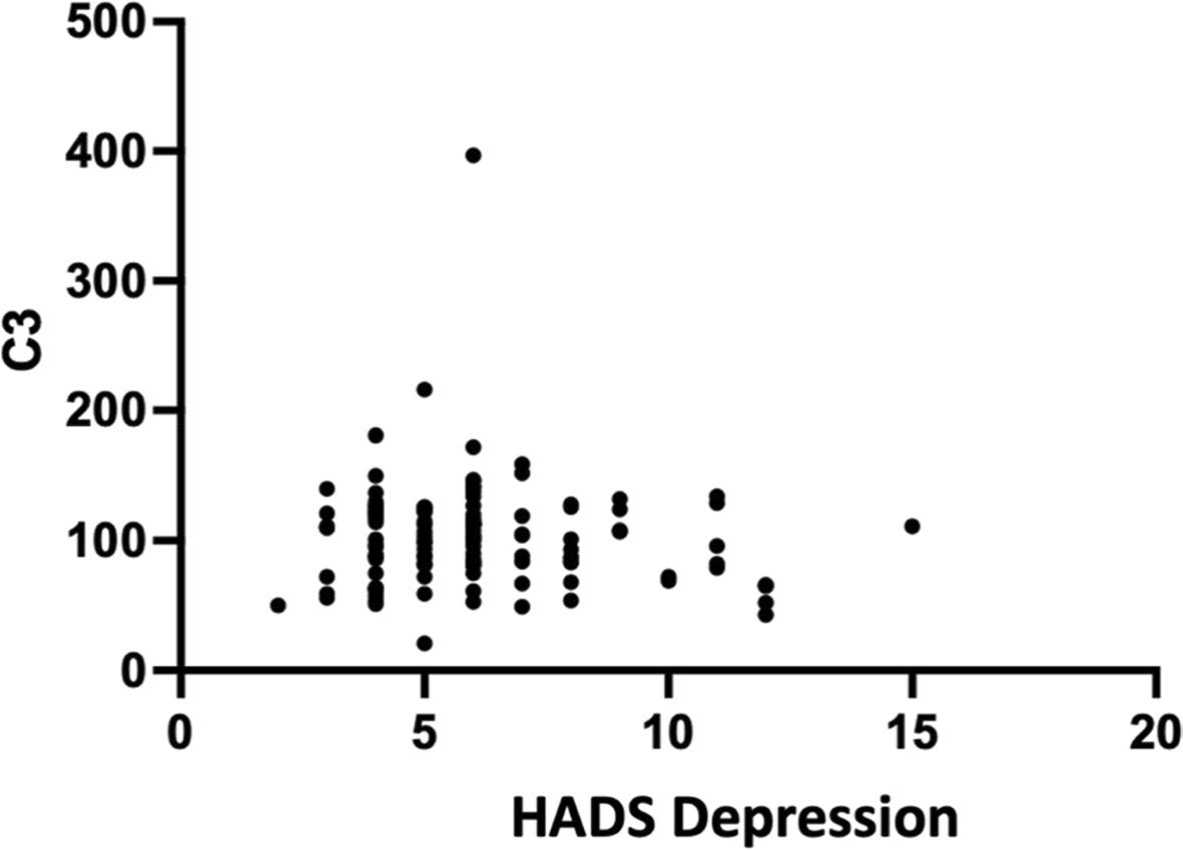
Correlation graph of HADS Depression with C3 levels

**Fig. 4:**
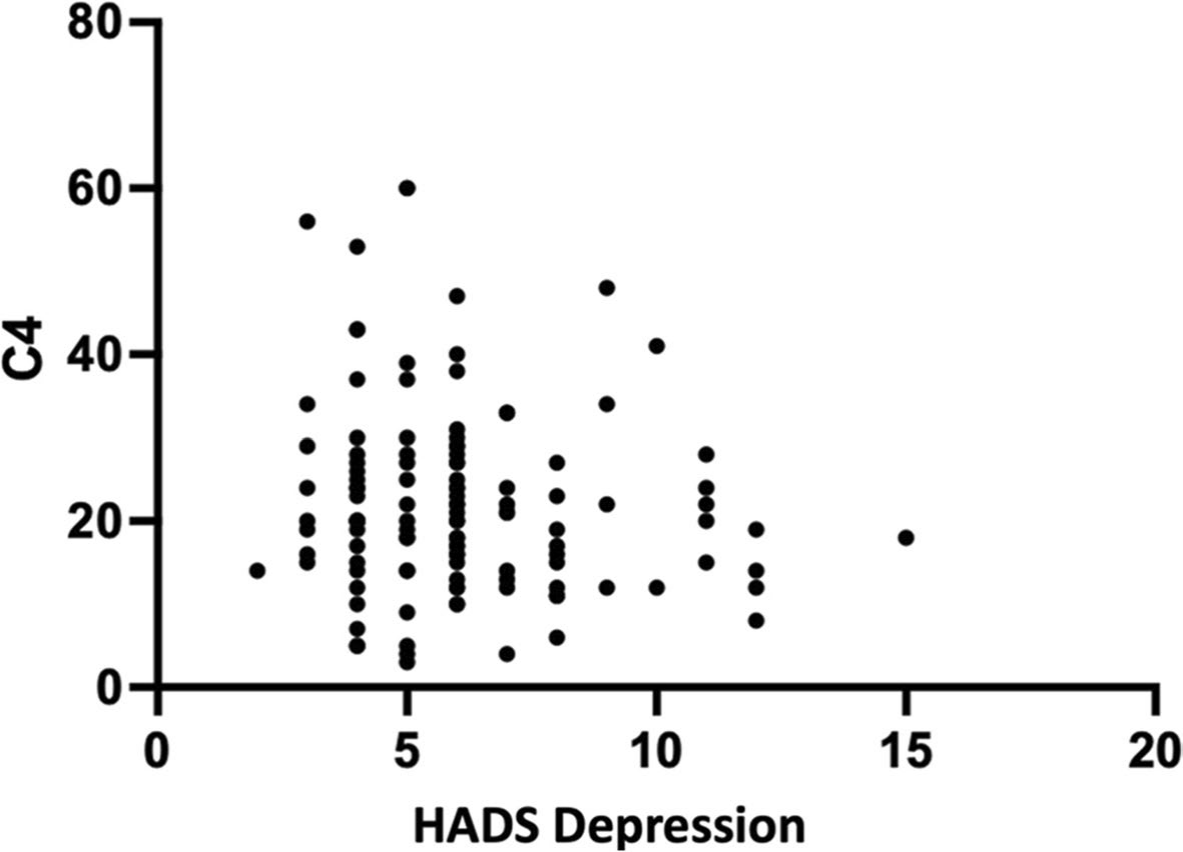
Correlation graph of HADS Depression with C4 levels

There were outliers in HADS-Depression and complement levels scatter plot. We still included them to analysis but we use non-parametric test. According to the Spearman correlation test results, there is not a correlation (*r*= -0.056; *p*= 0.546, 95%CI -0,23; 0,12) between depression HADS and C3 levels. However, the correlation between HADS depression and C4 levels was discovered to be extremely weak and not significant (*r*= -0.068; *p*= 0.461, 95%CI -0,24; 0,11). The correlation coefficients have a negative direction, as seen in Figs. 3 and 4.

### Correlation of HADS-Anxiety and HADS-Depression with MEX SLEDAI lupus activity

**Fig. 5:**
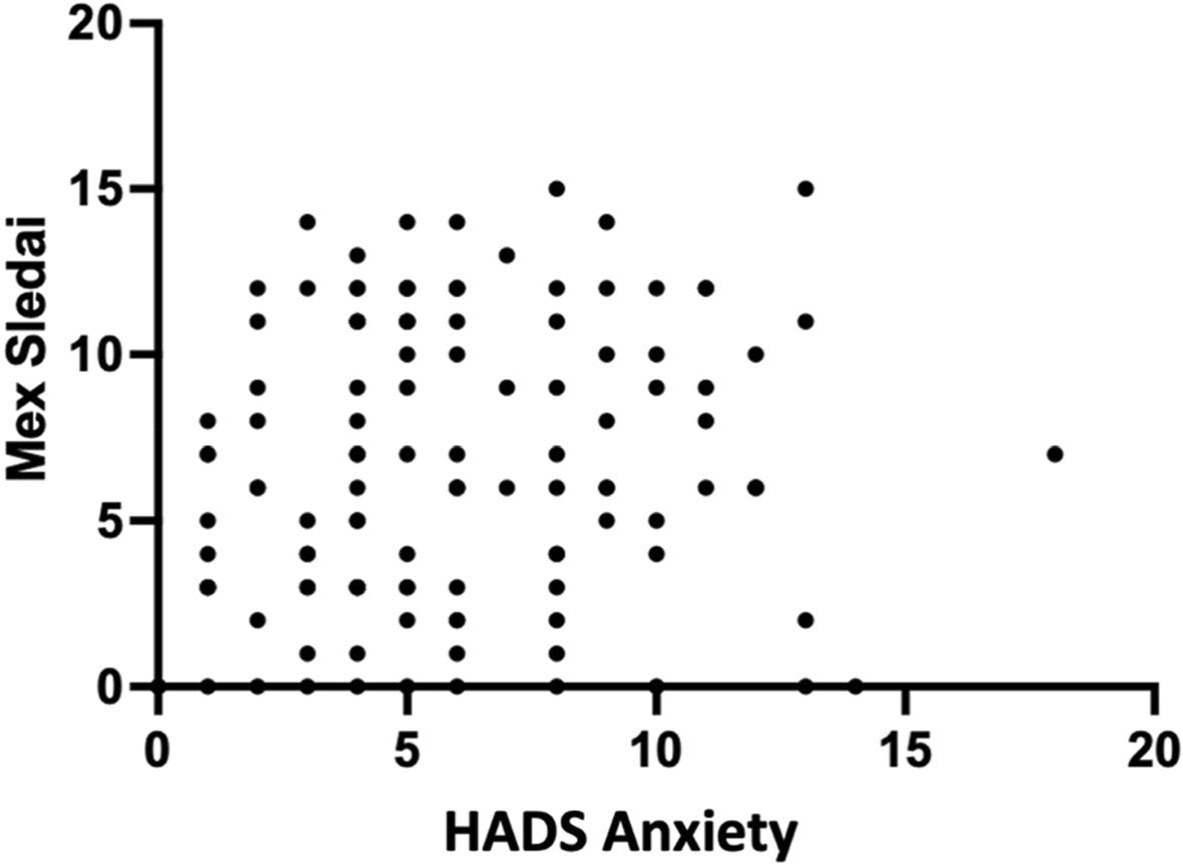
HADS Chart of Anxiety with MEX SLEDAI

**Fig. 6:**
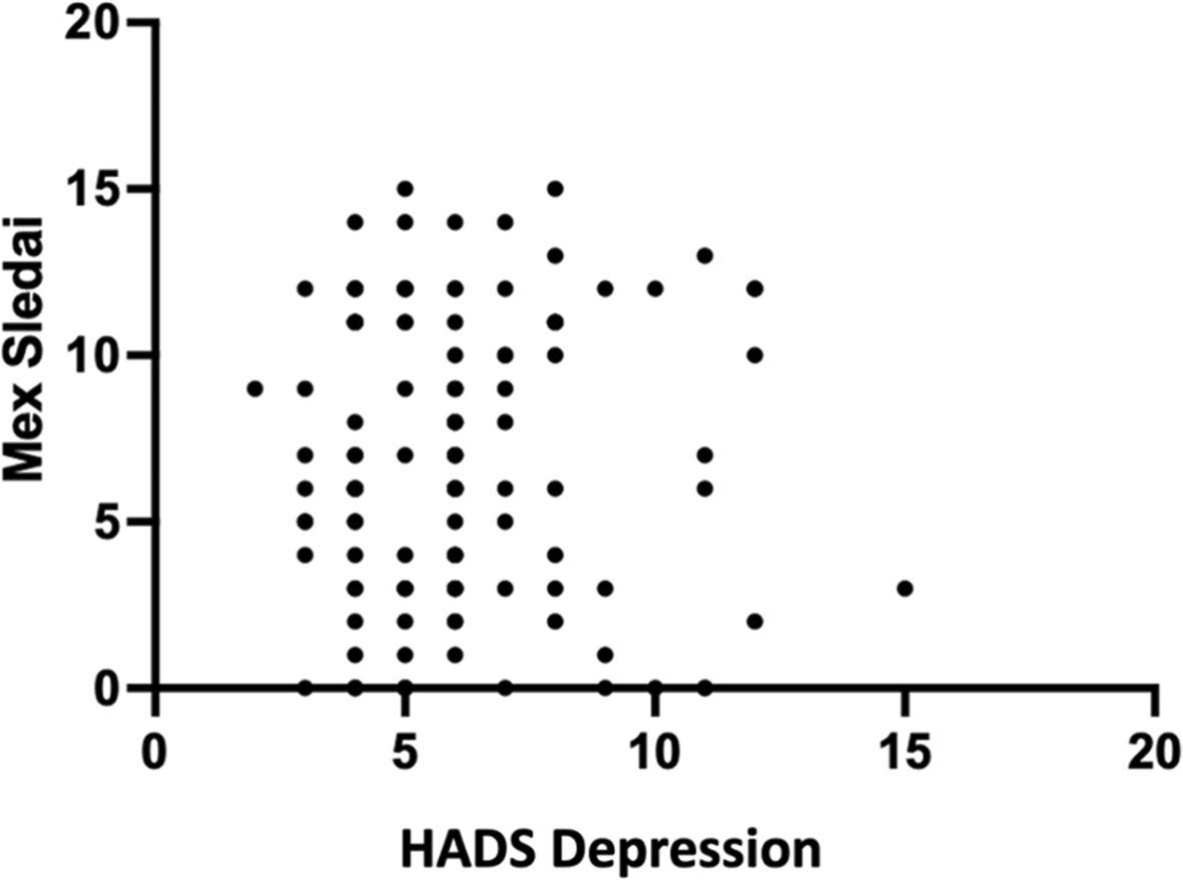
HADS Chart of Depression with MEX SLEDAI

A very small and insignificant positive connection (*r* = 0.06, *p* = 0.173, 95%CI -0,12; 0,24) was discovered between HADS-Anxiety and MEX-SLEDAI activity. While there was a very small and insignificant positive correlation between depression and lupus erythematosus activity (*r* = 0.31, *p* = 0.753, 95%CI 0,14; 0,46). Figures 5 and 6 show both outcomes.

### Multivariate analysis of depression and anxiety

#### Multivariate analysis of anxiety

As we already shown above, there is a significant correlation between complement levels to HADS-Anxiety score. In Table 4 below we resumed bivariate analysis of various variables to HADS Anxiety. All numerical variable has no normal distribution, so statistical analysis was done with non-parametric test (spearman for correlation, and mann whitney for mean difference). We found statistically significant correlation of C3 and C4 levels to HADS Anxiety. No other variables have significant correlation. But we include variables that has *p* < 0,2 in multiple regression analysis: steroid duration, MEX-SLEDAI, serositis, and azathioprine.

**Table 4:** Bivariate analysis of factors influencing HADS-Anxiety

VARIABLE	HADS Anxiety	*P* Value
Age (correlation)	-0,059	0,522
Steroid duration, correlation	-0,153	0,095
C3 levels, correlation	0,189	0,038
C4 levels, correlation	-0,185	0,043
Mex-SLEDAI	0,173	0,06
Marital Status, median (IQR)		
Unmarried	5 (4,5)	0,220
Married	6 (5)	
Divorced	8,5 (12,5)	
Organ Involvement,		
Mucocutaneous, median (IQR)		0,763
Yes	5 (4)	
No	5 (7,25)	
Musculoskeletal, median (IQR)		0,21
Yes	5 (4,25)	
No	7,5 (7,5)	
Hematology, median (IQR)		0,973
Yes	6 (4,25)	
No	5 (5)	
Kidney, median (IQR)		0,45
Yes	5 (4)	
No	5 (5)	
Serositis, median (IQR)		0,08
Yes	6 (4,5)	
No	5 (5)	
DMARD		
Azathioprine, median (IQR)		0,055
Yes	4 (3)	
No	6 (5)	
Cyclosporine, median (IQR)		0,558
Yes	6 (2)	
No	5 (4)	
HCQ, median (IQR)		0,838
Yes	5 (4)	
No	5 (5,5)	
Mycophenolate, median (IQR)		0,531
Yes	5 (5)	
No	5 (4,25)	

*C3* complement 3, *C4* complement 4, *DMARD* Disease-Modifying Antirheumatic Drugs, *HADS A* Hospital Anxiety and Depression Scale for anxiety, *HADS-D* Hospital Anxiety and Depression Scale for depression, *HCQ* Hydroxychloroquine, *Mex SLEDAI* Mexican systemic lupus erythematosus systemic disease activity

As we see from the Table 5, all variables have *p* value above 0,05. We tried to exclude variables with highest *p* value from the model, but it caused B value changes to more than 10%. After that we start from first model and tried to exclude second highest *p* value variable, but it also changes B value drastically. After trying that to all other variables, we cannot exclude any variable from the model. That model only has R square 0,097. It is also not fulfilling independency and linearity assumption.

**Table 5:** Multivariate analysis of factors influencing HADS-Anxiety

Coefficients^a^						
Model		Unstandardized Coefficients		Standardized Coefficients	t	Sig
		B	Std. Error	Beta		
1	(Constant)	7.182	1.191		6.030	.000
	Mexsledai	.066	.079	.081	.844	.400
	Lamasteroid	-.019	.011	-.155	-1.714	.089
	KadarC3	-.004	.008	-.042	-.430	.668
	KadarC4	-.030	.031	-.095	-.959	.340
	Azatioprin	-1.534	.837	-.169	-1.832	.070
	Serositis	1.548	1.228	.116	1.261	.210

^a^Dependent Variable: HADSanx

#### Multivariate analysis of depression

Like HADS-Anxiety above, we did bivariate analysis to HADS-Depression. As we can see in Table 6 below, only marital status has significant statistical difference. All analysis conducted using non-parametric test because the abnormality of data distribution. There are no variables have *p* value below 0,2. Because of that, we did not conduct multivariate analysis. Post hoc analysis conducted for marital status. We found statistically significant differences of HADS Depression score between married to divorced group and divorced to unmarried group. There was no significant difference of HADS Depression score between unmarried to married group.

**Table 6:** Bivariate analysis of factors influencing HADS-Depression

VARIABLES	HADS Depression	*P* Value
Age (correlation)	-0,098	0,286
Steroid duration, correlation	-0,007	0,94
C3 levels, correlation	-0,056	0,546
C4 levels, correlation	-0,098	0,287
Mex-SLEDAI	0,031	0,735
Marital Status, median (IQR)		
Unmarried	6 (3)	0,04
Married	5 (2)	
Divorced	9,5 (4,5)	
Organ Involvement,		
Mucocutaneous, median (IQR)		0,247
Yes	6(3)	
No	5,5 (5,5)	
Musculoskeletal, median (IQR)		0,629
Yes	6 (3)	
No	5,5 (3)	
Hematology, median (IQR)		0,864
Yes	6 (3,25)	
No	6 (3)	
Kidney, median (IQR)		0,367
Yes	6 (2)	
No	6 (3)	
Serositis, median (IQR)		0,847
Yes	5 (5,5)	
No	6 (3)	
DMARD		
Azathioprine, median (IQR)		0,478
Yes	5,5 (3)	
No	6 (3)	
Cyclosporine, median (IQR)		0,384
Yes	6 (2,5)	
No	6 (3)	
HCQ, median (IQR)		0,988
Yes	6 (3)	
No	6 (3)	
Mycophenolate, median (IQR)		0,564
Yes	6 (4)	
No	6 (2,25)	

*C3* complement 3, *C4* complement 4, *DMARD* Disease-Modifying Antirheumatic Drugs, *HADS A* Hospital Anxiety and Depression Scale for anxiety, *HADS-D* Hospital Anxiety and Depression Scale for depression, *HCQ* Hydroxychloroquine, *Mex SLEDAI* Mexican systemic lupus erythematosus systemic disease activity

## Discussion

Patients with HADS anxiety were detected in 35% of the patients in this study, whereas patients with HADS depression were found in 27%. This is consistent with the findings of Zhang et al., who reported that the prevalence of anxiety and depression with HADS was 40% and 30%, respectively, in SLE patients [6]. The 120 patients studied had a weekly steroid usage of 16 mg (0-28 mg). This indicates that the research subjects utilized low dosages of steroids to rule out the influence of medicines known to affect GABA [12]. The median lupus activity in this study was 6 (0-11), indicating that most individuals in this study had modest activity. As a result, mood-related NPSLEs produced by inflammation were ruled out [4].

Mucocutaneous involvement was the most common organ involvement in this study, followed by musculoskeletal and renal involvement. Previously, Osio-Salido et al. did an epidemiological analysis of SLE in Asia, where 52-98% of patients had mucocutaneous involvement, 36-95% had musculoskeletal involvement, and 30-70% had renal involvement. This is also consistent with the findings of Zayat et al., who discovered that the prevalence of musculoskeletal involvement in SLE patients ranged from 50 to 95% [14].

The C3 deficient level in the included cases reached 40%, while the C4 deficiency level was 12.5%, demonstrating how complement contributes to tissue damage and impacts prognosis in SLE patients. Tseng et al. discovered a strong negative connection between SLE activity and C3 and C4 (*r*= 0.552 and 0.276, respectively) in their study of SLE patients using SLEDAI [15]. This is comparable to what was done at Soetomo Hospital in Indonesia, where a correlation assessment between complement and Sledai was performed and it was discovered that low C3 in heavy activity reached 40% and low C4 levels reached 50.7% [16].

### Correlation of anxiety with C3 and C4 levels

In this study, a significant correlation was discovered between anxiety and C3 and C4 levels, with negative correlation values of -0.189 and -0.206. This score indicates a very weak relationship between the two. In SLE patients, this might be altered by a variety of circumstances. Currently, no research has been conducted to examine the correlation between anxiety and C3 and C4 levels, in contrast to prior studies that analyzed C5, which revealed greater activation in patients with severe depression, schizophrenia, and PTSD [17].

C3 is thought to play a role in non-immune neuronal activity, according to further research into the association between anxiety and complement and its control in the brain. C3 is implicated in the production of behavioral impairments and the modulation of synapses in the brain, according to the findings of a mouse animal trial by Shi et al. [18] C3 Knock Out mice demonstrated anxiety resilience and protection against age-related cognitive decline in learning ability and specialized memory, as well as an abnormally high number of synapses and improved synaptic efficacy in response to high-frequency bursts. Klos et al. found that C3 signaling via C3aR regulates synaptic function and neuronal dendritic shape. According to Cook et al., C3a, a cleaved form of C3, is involved in the recruitment of microglia to synaptically enriched regions. Thus, enhanced C3 signaling pathway activation in chronic inflammatory disorders such as SLE may contribute to anxiety-induced synapse loss, chronic stress, and alteration in synaptic connection in the PFC [19].

### Correlation of depression with C3 and C4 levels

There was not a significant correlation between HADS depression and C3 or C4 levels in this investigation. Isii et al. discovered greater complement C5 activation in patients with severe depression when tested in cerebral fluid compared to individuals with schizophrenia and PTSD. 15 Several research have been conducted to investigate the relationship between complement and the occurrence of depression. Data from mouse studies demonstrate elevated C3 expression in postmortem PFC participants, implying that C3 may play a role in the synaptic loss seen in depression patients. Increased C3 expression under chronic stress conditions, as well as reduction of stress-induced depressed behavior in C3 KO mice and C3aR KO mice, support the importance of C3 signaling in depressive behavior [20].

In a study by Luo et al. [21], it was discovered that peripheral levels of C3 and its active product, C3a, were significantly higher in drug-free patients with major depressive disorder (MDD) than in healthy controls (HC), whereas plasma concentrations of C1q and CRP were comparable in depressed patients and HC. In MDD patients, C3 levels were found to be related to Hamilton Rating Scale for Anxiety (HAMA) scores, although other clinical factors were not related to complement components and CRP [21].

According to the findings of Luo et al. [21], there was an increase in peripheral C3 and its active product, C3a, levels in drug-free MDD compared to HC. Although peripheral C3 in MDD has been studied extensively in the past, there have been very few research that look at both C3 and C3a at the same time. The findings of Luo's study add to the evidence for complement system activation in MDD patients, and they suggest that increased C3 may contribute to the pathogenesis of MDD via C3a. It should be emphasized that increased peripheral C3 in MDD has only been documented in a few studies and not in others. This disparity could be attributed to the diversity of MDD presentations and potential confounding variables that were not well controlled in these investigations. Overall, the findings of the Luo et al. study imply that raised peripheral C3 and C3a levels are pathophysiological changes that play a significant role in MDD, and C3 and C3a have the potential to act as biomarkers for MDD.

In Wei et al. [22] research of MDD patients, it was found that peripheral C4 was higher in MDD patients than in healthy controls. This also shows that C4 plays a significant role in the activation of the complement cascade, which is implicated in the development of inflammation. C4 is also expressed by neurons and is involved in synapse deletion. It is yet unclear if C4 is implicated in MDD via directly controlling neuronal function in the brain, but more research is needed. Antidepressants have long been known to lower inflammatory variables in MDD patients and animal models. Because inflammation can impact C4 plasma concentrations, the mechanism of anti-inflammatory effect exerted by antidepressant medicines may be related to C4 suppression. Previous research has suggested that antidepressants may reduce inflammation by regulating hypothalamic-pituitary-adrenal axis function or inhibiting immune cell activation. Although a lot of studies show an increase in peripheral inflammatory markers in MDD, there is also evidence of contradicting outcomes.

### Correlation of anxiety or depression with MEX SLEDAI

There was no significant relationship between anxiety or depression and lupus activity as measured by the MEX SLEDAI in this investigation. This finding contradicts the findings of other research, including one by Bai Ru et al. [23]. Furthermore, Jung Ju Yang et al. [24] discovered a link between depression and BDI scores and Lupus activity using SLEDAI (*r*= 0.253; *p*= 0.011). Several reasons may have contributed to the study's insignificant results. To rule out SLE-related inflammation, for example, very high SLE activity was not included as a participant. Furthermore, Jung Ju Yung et al. used SLEDAI, which has superior sensitivity where C3 and C4 are one of the elements that influence activity evaluation.

### Advantages and limitations

This study is recognized for being the first in Indonesia to examine the connection between mood disorders and complement levels C3 and C4 utilizing HADS. The cross-sectional study design of this investigation limits its long-term assessment of anxiety and depression. Furthermore, the study's findings included an anomaly in which one patient was determined to have significant lupus activity but a C3 level of 137. This can cause data analysis to deviate from the real results. Furthermore, this study employs HADS, where bias may come from response bias, as opposed to the Hamilton depression or anxiety examination, which uses a direct interview.

## Conclusion

In SLE patients, there was a significantly weak negative correlation between HADS anxiety and C3 or C4 levels. In SLE patients, there is not a significant correlation between HADS depression and C3 or C4 levels. There was no significant correlation between HADS anxiety or sadness and MEX SLEDAI lupus activity. Further research using a cohort design to investigate the connection of anxiety over time with complement levels and SLE activity is required. More study is being conducted on supportive psychotherapy, such as relaxation therapy, in SLE patients who are anxious, in order to examine both, complement and SLE activity.

## Data Availability

All data generated or analysed during this study are included in this published article.

## Abbreviations

BDI: Beck Depression Inventory
CK: Creatinin Kinase
CMV: Cytomegalovirus
DMARD: Disease-modifying antirheumatic drugs
HADS: Hospital Anxiety and Depression Scale
HIV: Human Immunodeficiency Virus
HAMA: Hamilton Rating Scale for Anxiety
HPA: Hypothalamic Hypopituitary Adrenal
HC: Healthy Controls
Mex SLEDAI: Mexican Systemic Lupus Erythematosus Systemic Disease activity
MDD: Major Depressive Disorder
NPSLE: Neuropsychiatry Systemic Lupus Erythematosus
PTSD: Post Trauma Stress Disorder
WHO: World Health Organization
